# Shape: automatic conformation prediction of carbohydrates using a genetic algorithm

**DOI:** 10.1186/1758-2946-1-16

**Published:** 2009-09-21

**Authors:** Jimmy Rosen, Laurence Miguet, Serge Pérez

**Affiliations:** 1 Bio Organic Chemistry, Utrecht University, Padualaan 8, 3584 CH Utrecht, Netherlands; 2 Centre de Recherches sur les Macromolecules Vegetales, CNRS, BP 53, F-38041 Grenoble, France

## Abstract

**Background:**

Detailed experimental three dimensional structures of carbohydrates are often difficult to acquire. Molecular modelling and computational conformation prediction are therefore commonly used tools for three dimensional structure studies. Modelling procedures generally require significant training and computing resources, which is often impractical for most experimental chemists and biologists. Shape has been developed to improve the availability of modelling in this field.

**Results:**

The Shape software package has been developed for simplicity of use and conformation prediction performance. A trivial user interface coupled to an efficient genetic algorithm conformation search makes it a powerful tool for automated modelling. Carbohydrates up to a few hundred atoms in size can be investigated on common computer hardware. It has been shown to perform well for the prediction of over four hundred bioactive oligosaccharides, as well as compare favourably with previously published studies on carbohydrate conformation prediction.

**Conclusion:**

The Shape fully automated conformation prediction can be used by scientists who lack significant modelling training, and performs well on computing hardware such as laptops and desktops. It can also be deployed on computer clusters for increased capacity. The prediction accuracy under the default settings is good, as it agrees well with experimental data and previously published conformation prediction studies. This software is available both as open source and under commercial licenses.

## Background

Saccharides linked to proteins and lipids cover a large fraction of the surface area of most cells. Many of these saccharides are involved in various recognition processes pertaining to immunity, signalling, adhesion, etc. To understand their biological function in detail it is necessary to have information about their three dimensional (3D) structure. Knowledge about the 3D structure of oligosaccharides also has medical applications, for example, in the design of vaccines targeted at surface saccharides of bacteria.

Experimental methods for 3D structure determination, mainly Nuclear Magnetic Resonance (NMR) spectroscopy and X-ray crystallography, are often not viable for studying oligosaccharide conformations in high detail. X-ray is commonly impossible because of the difficulty of crystallising oligosaccharides and NMR is often imprecise since most oligosaccharides yield few well defined NOE signals. This makes computational conformation prediction an attractive alternative method, either by itself or coupled together with NMR [1].

The common computational approach to 3D structure prediction is based on searching through the conformational space of the molecule in order to find low energy regions, i.e. conformers, which the molecule is likely to populate. This can be accomplished in many different ways, and several software packages have been developed for this task using a variety of different algorithms [2-5]. The main problem today in conformation prediction is the trade-off between speed and thoroughness. It is often important to perform a comprehensive general search of the conformation space to find all the important energy minima, while at the same time use as little computation time as possible. With Shape the user can choose a suitable trade-off between thoroughness and speed by altering the search parameters.

This work has primarily been targeted at modelling carbohydrates. It has been demonstrated to work very well also for other classes of biomolecules, but more testing and work is required before Shape can comfortably be presented as a general modelling tool for all types of biomolecules.

## Implementation

Shape employs a genetic algorithm (GA) as an efficient and simple search method, which over the years has been shown to perform well for search and optimisation problems in many areas of science and engineering [6]. GA strategies have also been successfully employed in ligand 3D structure prediction and design, as well as flexible docking [7-9]. A genetic algorithm is a heuristic method, and as such gives no guarantees that the global optimum, i.e. best the minimum energy conformation, will be found. It does however, when correctly parametrised, have a high probability of finding all important conformers while using relatively limited computation time. Commonly, Shape will find all interesting conformers of a pentasaccharide within a few hours using a laptop, while a decasaccharide usually takes about a day. Through the control parameters the user can select the trade-off between thoroughness, i.e. the probability of finding all interesting conformers, and the computation time requirements. Computer clusters can be used to speed up the calculations for large structures or libraries of many compounds.

The force field used for the energy calculations will have significant influence on the result of the conformation search [10]. The MM3 force field [11-13] is generally considered to perform well for carbohydrates, and has been chosen as the energy evaluation back-end. The MM3 force field is proprietary software and is not included in the Shape package. The user must have a functional MM3 installation in order to run Shape. MM3 can be obtained from the lab of professor N. L. Allinger [14]. Support for other force fields, primarily MM4 [15], Tinker [16], Gromacs [17], and Amber [18] is under consideration or early stage development. Support will be included in future releases as it becomes available, given enough time and funding.

The Shape software package is implemented in Java. The primary deployment platform is Linux, but other Unix systems are likely to work with little or no modifications to the source code as long as a Java 1.5 or newer jvm is available together with an MM3 binary. It can be used both from command line for easy scripting, and directly via the application programming interface (API) for more flexible and powerful use and inclusion in tool chains. Porting the package to other platforms such as Windows should be straight forward, provided that an MM3 binary is available. The interaction with MM3 is done via the mm3java library [19], which contains very little platform specific code. Users who has an interest in this should feel free to port the code directly or contact the authors regarding requests.

The choice of implementing in Java is based on integration aspects since most of the tool chains used in our labs are written in that language. The primary strength of Java is the human resources situation. It is relatively simple to recruit students and project engineers that have a reasonable experience with Java. From a technical standpoint the paired Python/C++ or Lisp can sometimes be better choices, but recruitment for those skills is unfortunately harder. One issue that is often raised when java is discussed is the performance loss compared to C++ and Fortran. Java is commonly a few times slower than C++ and Fortran, although not always. Shape spends more than 95% of the time in the MM3 energy evaluation back-end, so the potential performance hit from java is negligible.

The Shape package can be divided into a few separate units; molecular data and control parameter input, ga search together with the energy evaluation back-end, and result clustering. These tools can be used separately as well to some extent. Well known methods from various fields have been combined to create a useful and efficient tool chain. Each part of the software package is implemented in such a way that it performs efficiently for the task of molecular conformation search.

### Molecular data and search control input

Molecular data input is done through file import filters. The molecular structure files are read from a user specified source directory. The supported file formats currently include .pdb, Sybyl .mol and .mol2, and some .xyz variants. Care has been taken to support at least part of the existing plethora of dialects of the .pdb and .xyz file formats that exist in the wild. However, further work will always be needed in this area. Interconnection of object data structures with CDK [20] and Jmol [21] is available for those who wish to include functionality from those packages in their tool chain. Linking with JOELib [22] or Open Babel [23] data format libraries has been considered, but is not yet implemented.

Shape takes control parameters from a few separate files, corresponding to the various tasks it performs. Examples of all control files are provided in the package to simplify the setup, and the default configuration usually works sufficiently well for most users. A tiny configuration tool has been developed that uses a simple syntax in plain text files for configuration. By using java reflection, the same code can be used to configure most plain java objects and nested hierarchies.

The MM3 back end is provided through the mm3java [19] library. The configuration of the MM3 installation must be tweaked for most users, based on the provided examples. The MM3 configuration specifies the MM3 process environment, which force field parameter files to use, etc. For deployment on clusters, shared memory processing (smp) machines, and when deploying several parallel Shape processes, a multithreaded server configuration must be specified. This is done by changing the number of parallel MM3 workers that are to be started and by ensuring separate temporary work areas for parallel servers. Parameterisation of the GA will determine the efficiency of the search and the probability of finding all important conformers of the molecule. However, it is impossible to define one set of parameters that is optimal for all compounds. Depending on the complexity of the conformational energy hypersurface and the dimensionality of the search space, some GA search parameters settings will perform better than others. Several parameter sets are provided that work well for different classes of compounds and provides different speed vs thoroughness trade-offs. Parametrisation of the GA search is still under investigation and will be treated in depth in a future paper. In the future we hope to be able to enhance Shape with a tool that can automatically select or suggest suitable GA parameters without user intervention.

Clustering control parameters define how the clustering of results will proceed. Various clustering techniques, measurements, and weighting parameters are supported that describe how the results from the GA search will be clustered into families of conformers.

### The genetic algorithm

The genetic algorithm used in Shape is a straight forward implementation of a generational parallel population Lamarckian GA, supporting mutation, crossover, and migration. The genes are represented using real value direct encoding of torsion angles. All genes are stored in a single linear chromosome in an arbitrary order.

Genetic algorithms are inspired by nature and evolution. They generally consists of a few iterative steps which are looped until a termination criterion is met. The terminology is borrowed from biology. One specific conformation of a molecule is called an *individual*. An individual contains a *genome *describing some basic properties of the individual conformation. In our case the individual's genome corresponds to the torsion angles of that specific molecular conformation. Several individuals make up a *population*, within which the individuals will compete for survival. Individuals compete for survival based on their *fitness*, which in this case is calculated from their conformational energy. A low energy conformation corresponds to a high fitness score. A simple GA schema can be as follows:

1. *Initialisation: *One or several populations are initialised by creating a number of individuals with totally random genome, meaning highly random conformations.

2. *Evaluation: *All individuals in all populations are then evaluated. In our case each individual conformation is minimised by the MM3 force field back-end, and both the resulting geometry and final energy is stored. The minimised geometries are encoded to torsion angles and stored back into the genome of each individual. The fitness of each individual is calculated based on its final conformational energy, in comparison with all other individuals in the same population.

3. *Procreation: *All populations are then propagated to the next generation by creating a set of new individuals from the old ones. In this step there are several genetic operators at play, depending on the search parameters. *Mutation *is the operation of randomly changing one or more genes, i.e. torsion angles, in the individual conformation. *Crossover *is the process of creating a new individual by combining the genome from two or more parents. *Migration *is the method where one or more individuals move from one population to another.

4. *Termination: *The search terminates if one of several possible termination criteria has been met. Otherwise it continues, starting the next iteration with the evaluation step. Shape usually terminates when no significant improvement in the best individuals have been found for a sufficiently long time.

Lamarckian evolution is supported, wherein the minimised geometries of the individuals are inherited by their offspring. This creates a significantly faster convergence rate than a standard GA approach for this application. Mutation is parametrised either by frequency or probability. Two versions of mutation operators are available, either totally random mutation, which sets a torsion angle to a random value, or an additive mutation, which modifies a torsion angle with a random amount. Crossover is supported by inheritance from two or more individuals with the probabilities of multiple parents and weight of inheritance between parents are determined by the search parameters. Different scoring and selection methods are available for selecting parents for the next generation of individuals. The most successful is to employ a ranking based scoring model and a roulette wheel based selection model.

The GA can be set to use either one or several parallel populations, with specified sizes. Migration between populations is possible. We have noticed that in general, especially for small compounds, one large population is sometimes more efficient than several smaller ones. This is consistent with other published results [24]. However, for larger molecules with a more complex energy hypersurface, it can be difficult to find a global minimum with only one population. General recommendations and example parametrisation for large complex structures will be made available when sufficient data has been collected. The provided standard parameters work reasonably well.

Shape has initially been developed for conformation prediction of carbohydrates. It searches primarily the rotational torsion angles of the compound. Ring structures are detected and high energy ring conformations are ignored as they impart too high energy on the conformers, and are thus generally not seen in nature.

### Clustering

The GA conformation search produces a large number of conformations. All evaluated conformations are stored as they provide the full evolution trace. The results are then clustered by a very simple clustering algorithm: All conformations are sorted by order of increasing energy. The first cluster is then seeded by the lowest energy conformation. The cluster is grown by adding all conformations that fall within a specified distance from the seed centroid conformation. The next cluster is then seeded with the lowest energy conformation that is not already assigned to a cluster, and subsequently grown to encompass all non assigned conformations within the specified distance. This continues until a certain percentage of the conformations have been assigned to clusters.

This is a highly simplified algorithm that does not allow any adaptive movement of the centroids. It is however fast, with average algorithmic complexity significantly faster than O(c·n), where c is the number of clusters and n is the number of conformations. This clustering approach has turned out to be efficient in locating all major conformers in the comparative studies we have performed. The down side is a possible production of small false positive clusters, i.e. sets of conformations that just happened to be slightly outside the cutoff distance from previous centroids.

A few different combinations of distance measurements and weighting formulas are supported. The primary distance measurement is the RMSD of atomic positions. Different weights can be applied to the atoms in few ways. All atoms can be assigned have equal weight. Atoms below a certain mass can be discarded. Atoms can also be weighted based on their mass, either linearly or by the square root. The second distance measurement implemented is based on RMSD of the torsion angles. The torsion angles can be weighted evenly, or based on one of several expressions of the total mass of the smallest subgraph of the molecule that is separated by the torsion angle. The available measurement and weighting schemes allow for flexible distance measurements for the result clustering.

## Results and discussion

One of the primary goals of the Shape development was to create a tool that is very simple to use for the end user. Even for users who know nothing of molecular modelling. The current release of Shape meets those goals very well. Using the Shape software is exceptionally simple. The end user simply copies the molecule files he or she wants to investigate to the queue directory, and can then sometime later pick up the finished results in the results directory. The default settings of the program will perform well for the common use situations, but users who want more control over the search procedure can change the settings. Shape is controlled through parameter files where the user can change the behaviour of the software to suit his or her needs and preferences. A selection of tested parameter sets are available for various general molecular target areas.

Shape can be run as a daemon or a foreground process, or via the API inside an existing process.

Molecules are handled sequentially based on the timestamp of the file, with oldest first. Hence it is possible for the user to dump molecular files into the source directory of a running Shape process and they will be treated in an orderly fashion. It is of course also possible to run several instances of Shape in parallel in the same logical machine space regardless of whether it is one single machine or a cluster.

Shape performs well on a modern laptop when analysing single molecules or small libraries, and can be deployed in a cluster environment for prediction of large libraries of compounds. The software scales almost linearly up to reasonably sized clusters depending on the GA control parameters in use. Shape has been used to predict the 3D structure of over 400 bioactive oligosaccharides, ranging in size from disaccharides to tetradecasaccharides. Running on one single desktop machine it was possible to provide good conformation prediction data in less than one year for this library of compounds. The resulting data is part of the 3DBAO curated database of bioactive oligosaccharides and will be presented in a future paper. To give a good picture of the search performance of Shape it has been used to perform a series of studies on previously published conformation predictions of oligosaccharides performed using other conformation search tools. The influence of the back end force field as well as the simulation environment is significant for the calculation of relative energies between conformations. To reduce this problem, and allow for comparisons, all the case studies presented below have originally been done using the MM3 force field for energy calculations, in vacuum and with a high dielectric constant. Thus the focus is solely on the performance of the search engine, and not on the energy evaluation method.

The results are often not *exactly *the same between different studies, or even between successive runs with Shape. This is mainly because not enough computation time is allowed for the total optimisation of minute details such as the orientation of a few hydroxyls or miniscule changes to glycosidic torsion angles. For small structures, di- and trisaccharides, this is usually not evident since the default search parameters are a bit too thorough. In those cases the energy difference between global minimum conformations found by different runs can be at or near zero, corresponding to more or less identical conformations. For larger system the default parameters are less thorough in order to save time. Minor differences are commonly seen in those cases. For pentasaccharides there are usually one or two kJ/mol difference between found global minima. For decasaccharides the difference is less than 10 kJ/mol, i.e. less than 50 J per atom per mole. This effect can be reduced by increasing the thoroughness of the search, with a higher computational cost. Below follows comparisons with some previously published modelling results. Those studies have originally been carried out using other modelling tools, all with MM3 as the energy evaluation back-end.

The *Shigella Dysenteriae *type 2 O-antigen consists of many repeats of one pentasaccharide subunit. The structure has been predicted several years ago [25] using a grid based systematic search approach [3] and experimental data is available that supports the original prediction [26]. When applying Shape to this oligosaccharide all major local minima are found within a few hours running on a single desktop machine.

The global minimum conformation found is generally within 2 kJ/mol of the published 3D structure. The conformation clusters found by Shape encompass all major conformers that are found in the original study.

The *Shigella Dysenteriae *type 4 and *Escherichia coli *159 O-antigens both consist of repetitions of two similar pentasaccharide subunits. Conformation predictions of these oligosaccharides have been published in a study aimed at explaining the immunological cross reactivity between the species [27]. Both oligosaccharides present sterically constrained 3D structures with three important minima. Shape consistently finds all important minima in a few hours to within 2.5 kJ/mol energy difference and the clustered results provide good sampling around all minima found in the original study.

The conformation of the *Burkholderia cepacia *exo-polysaccharide has been predicted [28] using the GLYGAL [4] conformation search tool. To correctly predict the conformation of the polysaccharide one must take extra care since long range interactions exist between the repeating units of the polymer. The basic simulation system consists of the repeating unit heptasaccharide, but additional upstream saccharide units must be added for the search to arrive at the correct global minimum conformation. This results in search units of sizes between seven and 14 sugars. The low energy conformations of the heptasaccharide simulation systems are found by Shape within five to ten hours. The larger systems require up to a couple of days to terminate with good descriptions of all interesting conformers.

In the original study, the conformation search using GLYGAL took several weeks on a 10cpu cluster. With Shape the same study takes only a few days on a single desktop machine with the same cpu speed that was used in each node of the cluster in the original study. This is a significant improvement in efficiency. The resulting global minimum predictions are within 5 kJ/mol difference between the GLYGAL and Shape results, and the clusters around the interesting conformers are well sampled. GA parametrisation of these large compounds of more than 10 sugars still requires improvement due to lack of data, and it is likely that further improvements will be achieved for most target systems.

A conformation prediction study of a large tetradecasaccharide, the uromodulin O-glycan, has been published by Cioci et.al. [29]. The study makes use of the CICADA [2] conformation search tool. Cioci have kindly provided the original geometry files with the global minimum found in the study. CICADA performs a deterministic guided systematic search through the conformation space by following low energy paths on the energy hypersurface. This is a thorough and efficient approach. The uromodulin O-glycan is the most difficult saccharide we have tested with Shape. The conformational space is complex and the global minimum conformation has a distinctly folded shape. A thorough search of the conformational space is necessary before the global minimum conformation is found. The search terminates in a few days on a dual 1.8 GHz Athlon MP desktop computer. The original study by Cioci used three cpu months on a MIPS R14000. The 1.8 GHz Athlon MP cpu has a benchmarked molecular mechanics computation capacity that is approximately twice that of the MIPS R14000. This comparative study has shown that Shape performs well, both in speed and accuracy, also on large carbohydrates with well defined low energy conformations and a complex folded shape.

A more thorough analysis of convergence behaviour under various search parametrisations is under investigation and will be presented in a future paper. We will also study the application of Shape to target molecules from other areas of biochemistry, such as small peptides and synthetic compounds.

## Conclusion

This paper describes the successful use of Shape for conformation search of oligosaccharides up to a considerable size, which covers most of the known biologically active oligosaccharide compounds. The possibility to deploy Shape on a wide range of hardware enables its applicability to a variety of usage situations ranging from scientists who wants to find conformations of a single or a few compounds using their laptop, to a laboratory or company who wants to process large libraries of compounds fast on a Linux cluster.

Shape performs well in comparison to other existing software tools for conformational search of oligosaccharides. The main limitation on performance and applicability is the current lack of support for force field back-ends other than MM3. Work is underway to improve the situation, and alternatives will be available in future releases. Various improvements in control parametrisation and search efficiency is under development and will be made available in future releases.

Shape is primarily released as open source software under the GNU General Public License (GPL) [30]. The main reason for this is to facilitate the integration of this software into existing computational pipelines and work procedures directly by the power user. Open source also makes it simpler for the power user community to modify the behaviour of the software while stimulating the back propagation of changes to the software maintainer. Improvements and extensions built by the community can thus be included in future releases and the quality and capabilities of the code may increase faster than in a closed source environment.

To facilitate the use for commercial users, the Csol corporation can distribute the package under alternative commercial licenses, and can also provide commercial support upon request.

It is our hope that the Shape software package will be useful for a wider audience in the scientific community. We welcome comments, discussions, requests, and collaborations for the future development path of the project.

## Availability and requirements

• **Project name: **Shape

• **Project home page: **http://sourceforge.net/projects/shapega

• **Operating system: **Linux

• **Programming language: **Java 1.5 or higher

• **Other requirements: **MM3

• **License: **GNU GPL and commercial license

The Shape software has only been tested under Linux. It is likely to run on other platforms that support java, provided that an MM3 binary is available. Some minor adjustments may be required.

Commercial licenses are available from Csol [Rörmöstvägen 96, 434 93 Vallda, Sweden], as an alternative for those who do not wish to use the software under the GPL license.

Version 090213 of Shape is available as an additional file [see additional file 1]. Future versions will be made available from the project homepage on SourceForge as they are released.

## Competing interests

The author Jimmy Rosen has financial interests in the Csol corporation which can provide commercial licenses and support for the Shape software package.

## Authors' contributions

J.R. has done software design and implementation. L.M. has done testing and result verification. S.P. has done test compound selection and curation.

## Supplementary Material

Additional file 1 **Shape version 090213**. The complete shape distribution. Click here for file
