# Supplementary material for: Shape: automatic conformation prediction of carbohydrates using a genetic algorithm
Source: J Cheminform. 2009 Sep 21;1:16. doi: 10.1186/1758-2946-1-16 (PMC2820494; doi:10.1186/1758-2946-1-16)
Supplement: Additional file 1 — Shape version 090213. The complete shape distribution. [file 1758-2946-1-16-S1.TGZ › shape.release.090213/manual/configuration.html]

# Configuring Shape

Shape behaviour is controlled through the configuration files. The \*.config files specify the necessary parameters that determine how the program behaves. The configuration parameters include locations of files and work areas, selection of algorithms to use, settings that determines algorithm behaviour, etc. Most of these settings have good default values for general use.  
The names of the configuration files provided here are the default names. They can be changed by providing alternatives either on the command line, for the main Shape configuration file, or in the shape configuration file itself, for the other configuration files.  
  
It is strongly recommended that you use an editor that have good color coding and set the editor's file filters to treat all \*.config files as shell script or configuration files. That way it is very easy to see which lines are comments and documentation, and which lines are parameter settings. It is also important that the editor has parenthesis and brace matching to make it easier to avoid making mistakes with nested braces and parentheses.   
  
The syntax of the configuration files is generally very simple, but can get a bit complex when configuring the more flexible parts of the Shape package. The syntax has been chosen to simplify the configuration of many different programs and algorithms using the same syntax.   

- Lines that start with a "#" hash mark are comment lines and are ignored. Comments cannot be placed after other parameters, and must occupy a line of their own.
- Value parameters are specified "parameter" "value" pairs, with each pair on one line:   
  parameter value  
  with the parameter and the value separated by one or more spaces. There should be no quotes or citation marks around neither the parameter name string nor the value string, and do not use "=" equal signs to separate the parameters and their values.
- All config files for the Shape program suite are case sensitive. rootDir, RootDir, rootdir, ROOTDIR are all different, and in this example, only rootDir is in correct case. This follows the de-facto java programming standard of variable case naming. The basic idea is that all variable names are written as oneWordOnly never with hyphens spaces or underscores. Names that are made up from more than one word will have the leading word in lower case, and all following words with the first letter in upper case. E.g. smallBirdsNest, redBall, fireAnt.
- Configurations can be nested. Some parameters doesn't take regular values, but requires whole blocks of configuration. This is to make it easier to swap in code modules through the configuration, without having to write different configuration code for each class or recompile the software. In those cases a configuration block is surrounded by curly braces "{}" like this:  

  ```
          baseParameter full.qualifier.to.ExampleClass {
              subParameter1 value1
              subParameter2 value2
          }
  ```

  In this example the subParameters are properties of the class ExampleClass. The parameter baseParameter gets assigned an object of the ExampleClass type, and that object is then configured with the subParameters.   
  This also makes it easy to write and switch in your own code if you are unhappy with the current behaviour and limitations.  
  This nesting requires that the full java qualifier of the class is provided. This is onerous but important for allowing of swapping in code from external packages.   
  This style of configuration has been chosen to facilitate development and make it easer for the user to extend the functionality of the system. For this aspect the immediate "ease of use" has been given lower priority than the extensibility of functionality. When changing the class qualifiers of these block statements you are not just changing a parameter value, you are in fact plugging in and out different parts of the software in runtime.

  
Shape requires several configuration files. The configuration has been split up into different files to make it fast and easy to write and maintain different behaviour between projects. The primary configuration file "shape.config" determines which other configuration files will be used. Therefore it is very fast and easy to write different search or clustering behaviours and link in to a shape behaviour by switching out the file names in the primary config file.  
 This also makes it easy to keep different configuration files for early stage quick screening, and more thorough searches, when dealing with large libraries of molecules.   

1. **shape.config** is the primary configuration file for the shape package. It mainly provides file locations and work directories.
2. **shape.server.config** contains the configuration for the job distribution server. It decides how many concurrent jobs should be run and the temporary work locations.
3. **shape.search.config** specifies how the conformation search will be performed.
4. **shape.cluster.config** determines how the results will be clustered. Which algorithms will be used, tolerances, measurements, etc.
5. **shape.mm3.config** describes the MM3 environment Shape will use to perform the energy relaxations.
