# Supplementary material for: Shape: automatic conformation prediction of carbohydrates using a genetic algorithm
Source: J Cheminform. 2009 Sep 21;1:16. doi: 10.1186/1758-2946-1-16 (PMC2820494; doi:10.1186/1758-2946-1-16)
Supplement: Additional file 1 — Shape version 090213. The complete shape distribution. [file 1758-2946-1-16-S1.TGZ › shape.release.090213/manual/output.html]

# Understanding Shape console output

Shape doesn't yet have a built in logging facility. It is planned for future versions of the package, but of low priority since the excellent tool "screen" can be used to both run the shape tool in a decoupled background console and provide logging for the output at the same time.   
The "screen" utility is highly recommended to know, even if you never use shape, since it has many other uses.   
  

### Startup

When shape starts up it will output some basic startup messages declaring the current configuration in use, and such things.

```
Shape.main() commencing. Shape program starting.
    Beginning setup
    using root directory:                 /home/jiro/projects/shape
    using main configuration file:        shape.config
    using mm3 configuration file:         shape.mm3.config
    using search configuration file:      shape.search.config
    using server configuration file:      shape.server.config
    using clustering configuration file:  shape.cluster.config
    using srcDir:                         /home/jiro/projects/shape/src
    using outDir:                         /home/jiro/projects/shape/out
    using errDir:                         /home/jiro/projects/shape/err
    using tmpDir:                         /home/jiro/projects/shape/tmp
    using sleep interval:                 10000ms
Shape.run() commencing -- initializing subsystems.
    Starting server
    Setup complete, entering work mode
    Looking for data source files to work with.
```

Above is a typical startup console printout. It declares that the program is starting from the .main() entry point, then lists the file configuration setup of which configuration files are being used, and which directories have been selected for input and output.  
After this it initialises the subsystems and starts the server.  
  
When the startup is complete Shape will either find a molecule in the source directory and go to work, starting the conformation search, or it will go to sleep for a while and try again later.   
  
When a molecule has been found Shape will first try to run the molecule once through MM3 to see if it can be managed by the MM3 back end.

```
================================================================================
New source file found: "/home/jiro/projects/shape/src/disacch.mol" attempting MM3 validation.
File "/home/jiro/projects/shape/src/disacch.mol" passed MM3 validation
```

Above is typical output from the MM3 validation step.  
  

### Search evolution

Once a molecule source file has been found and verified the conformation search can start.  

```
Starting conformation search on file: "/home/jiro/projects/shape/src/disacch.mol"
    setting up the search engine
testing the progenitor molecule
    configuring search enging
    starting conformation search
```

Shape starts up the search engine and runs a minimization of the progenitor conformation to make sure it can be used for evolution. Then the conformation search iteration begins.   
  
For each generation the search will print an evolution snapshot:  

```
evolution snapshot:
    Individual 472: g=11, e=29.9271, f=0.354, is ancient
    Individual 490: g=12, e=29.9269, f=0.500
    Individual 491: g=12, e=39.3375, f=0.044
    Individual 492: g=12, e=31.8756, f=0.062
    Individual 493: g=11, e=30.0519, f=0.177
    Individual 494: g=12, e=30.4646, f=0.125
    Individual 495: g=12, e=29.9264, f=0.707
    Individual 496: g=12, e=30.032, f=0.250
    Individual 497: g=12, e=29.8555, f=1.000
    Individual 498: g=12, e=31.8408, f=0.088
Selected parents : 494 490 => child: 499
Selected parents : 497 => child: 500
Selected parents : 495 496 => child: 501
Selected parents : 498 490 497 491 => child: 502
Selected parents : 497 495 497 490 => child: 503
Selected parents : 490 495 472 497 => child: 504
Selected parents : 496 497 495 490 => child: 505
Selected parents : 497 490 => child: 506
Selected parents : 494 490 497 => child: 507
```

For each population Shape will list the individuals, their generation (g), energy (e), and fitness score (f).  
Then it will list the breeding selection, where it can be seen which parents are used to generate offspring in the next generation. A list of selected parents are used to generate each child.  
  
Shape will also perform a convergence check of each population at the end of evaluating each generation of that population.  

```
covergence check for population 2 at generation 13
old: 30.3724, min: 29.8446
limit: -0.5, diff: -0.5277999999999992
convergencewindow: 30.372, 29.930, 29.927, 29.856, 29.845,
```

Where it lists which population and generation it is checking, then the energy of the "old" first best individual in the convergence window, along with the minimum energy found in the window. Then it lists the current energy limit and current energy difference in the window. When the diff is higher than the limit, then the search will eventually terminate. Then it prints all the best energies of each generation in the convergence window.  
  

### Clustering output

The printout of the result clustering is mainly just a progress indicator.  

```
--------------------------------------------------------------------------------
Starting clustering of search results.
The cluster result output directory "/home/jiro/projects/shape/out/disacch.mol/cluster.result" already exists. The file path is being removed by force to make room for the new results. Deleting "/home/jiro/projects/shape/out/disacch.mol/cluster.result" recursively ... done
Clustering: LECFunction.cluster() is forming cluster 0
Clustering: LECFunction.cluster() is forming cluster 1
Clustering: LECFunction.cluster() is forming cluster 2
Clustering: LECFunction.cluster() is forming cluster 3
Clustering: LECFunction.cluster() is forming cluster 4
Clustering: LECFunction.cluster() is forming cluster 5
Clustering: LECFunction.cluster() is forming cluster 6
Clustering: LECFunction.cluster() is forming cluster 7
Clustering: LECFunction.cluster() is forming cluster 8
Clustering: LECFunction.cluster() is forming cluster 9
Clustering: LECFunction.cluster() is forming cluster 10
Clustering: LECFunction.cluster() is forming cluster 11
Clustering: LECFunction.cluster() is forming cluster 12
Clustering: LECFunction.cluster() is forming cluster 13
Clustering: LECFunction.cluster() is forming cluster 14
Clustering: LECFunction.cluster() is forming cluster 15
Clustering: LECFunction.cluster() is forming cluster 16
Clustering: LECFunction.cluster() is forming cluster 17
Clustering: LECFunction.cluster() is forming cluster 18
Clustering: LECFunction.cluster() is forming cluster 19
Clustering: LECFunction.cluster() is forming cluster 20
Clustering: LECFunction.cluster() is forming cluster 21
    converting clusters to PDB format...
Clustering completed without errors.
Handling of molecule disacch.mol has completed.
```

In the printout above, from a very short search, the Shape clustering tool says it starts up the clustering facility, then deletes the old clustering results that were found in the results directory. While shape will retain old search data and just add to the search information when a molecule is run several times, the old clustering results are deleted by force when a new result clustering is initiated. This is because there is no use having old clustering results lying around since they are obsolete when new search data has been found.  
Shape clustering then prints the progress as it is forming the clusters.  
Then converts all individuals to PDB format.  
Then the clustering is complete and exits.  
  
At this point Shape will go and look for new molecules in the source directory, or go back to sleep if none can be found.  
  

### Error messages and finding serious problems

Shape will every now and then encounter serious problems with for example input, configuration, or MM3. Unfortunately this is an area which is not so well developed yet. Some sections of the Shape code package contain more error handling code than actual business logic, but still the error handling and especially error reporting need to be significantly improved. For that we do apologize.  
When encountering errors, Shape will usually spew a java stack trace, along with some, hopefully, helpful comments and explanations. If this is enough for you to figure out what is wrong, then great, correct the error and try again. If it is not, then please write a bug report and contact the developers. The instructions for filing bug reports can be found in the end of the introduction section.  
  
If an error seems to have occurred:  
First try to see if shape is still running. If the Shape console is back to command line prompt, then Shape has indeed died and needs to be restarted. Otherwise wait a little and see if the program continues to print any understandable "normal" operations output. Some errors are recoverable within Shape, but will still produce copious error message reporting to the console.  
Secondly try to restart shape with the same input and see if the error is reproducible. It is very good if the error shows up again, in the same way and time. Then either fix the error if you can, or file a bug report.   
  
Typical problems that Shape can encounter are problems with the configuration, problems with understanding the molecule source file, and problems with running MM3 on the source file molecule.  
Configuration problems can be tricky to nail down since the error reporting for configuration files is very complex and also far too verbose. This is a problem with the reflective design of the configuration process, and will hopefully be improved in future releases.  
It is recommended that you check the spelling and case in the configuration files, that you are directing Shape to use the correct files, that you have the MM3 licensing set up correctly if you are using your own MM3 environment, etc.  
Shape will sometimes have problems reading molecule source files. The input file format readers are still rudimentary, but improves as the developers get more examples of molecule files to test with. So, please send us the files that Shape have problems reading.  
MM3 will sometimes have problems running minimization on a molecule. This is usually due to limitations in the MM3 autotyper, and outside of the Shape extra typers expertise. Again, if you can please send us these molecules we can try to improve the Shape extra typer. However, some molecules will not work with MM3, and some molecular graph patterns have been shown to be too slow for MM3 to manage, and are thus treated as too problematic to run with MM3. This problem will hopefully improve in future releases when Shape can use Tinker or other molecular simulation back end software packages, other than MM3.
