# Supplementary material for: Shape: automatic conformation prediction of carbohydrates using a genetic algorithm
Source: J Cheminform. 2009 Sep 21;1:16. doi: 10.1186/1758-2946-1-16 (PMC2820494; doi:10.1186/1758-2946-1-16)
Supplement: Additional file 1 — Shape version 090213. The complete shape distribution. [file 1758-2946-1-16-S1.TGZ › shape.release.090213/manual/introduction.html]

# Shape, introduction

This is an introduction to the basic functionality and operation of the Shape program package. Shape is meant to be *very* simple to use once it is installed and set up. It requires no knowledge of modeling to run. Below are the tree basic steps to using Shape to find the low energy conformations of your molecule.   

1. Draw your molecule and save as a .pdf file.
2. Copy the molecule file to the Shape source directory.
3. Get the result from the shape output directory when they are finished.

Shape is primarily meant to run in the background as a daemon service, and works without any manual interaction. It monitors the specified source directory for incoming files. When a file is found it tries to search the conformational space of the molecule to find all interesting minimum energy conformations. When it is finished it writes the results to the output directory and clusters all the found conformations into different conformer groups based on 3D structure similarity. Below are the three usage steps in more detail:   

1. Draw your molecule in 3D, or convert it to 3D, then save as a 3D molecule drawing in the common .pdb file format. Shape understands .mol and .mol2 and some other formats as well, but .pdb is usually the best bet. If you don't have a program for this then pymol, ghemical, etc are free to download. Commercial alternative include Corina, Confort, Sybyl, etc.
2. Copy the .pdb file to the "src" directory of the Shape installation. Shape will process all provided source .pdb files in order of arrival. Actually it is oldest file first, but that usually means in order of arrival.
3. Once Shape is finished with the molecule you can collect the results in the "out" directory. The results are all compiled under a directory with the same name as the molecule source file that was processed.

All the results from the conformation search can be found in the output directory, normally called "out", under a subdirectory named after the molecule source file. This directory contains a lot of information. The entire search procedure is in fact stored here. What people usually want is just a quick view of the best results. This can be obtained in the cluster.result/cluster.centroids directory. Below are two bash commands to view the ten best local minimum conformations found, and the index of all the primary local energy minima.

```
    > pymol cluster.result/cluster.centroids/pdb/c*[0-9].*.pdb 
    > nedit cluster.result/cluster.centroids/index
```

The first line will open the 10 best local energy minima in the pymol viewer, and the second line will open the index of the best local minima in the nedit editor. You should of course change this to whatever software you normally use for this kind of work.   
  
  
(fig 1) Above is an example of a very quick search of a small trisaccharide. The first command copies the molecule file "triglc.pdb" to the "src" directory. The shape daemon then performs the search. The following commands just list the different levels of output directories. The cluster centroids are ordered by increasing energy in the "cluster.result/cluster.centroids/pdb" directory, and they are indexed with relevant data in the "index" file.   
  
  
(fig 2) The figure above are the top six cluster centroids from the output shown by pymol. The lowest energy conformation is shown as thick sticks and the rest as lines.  
  
The limitations of Shape are mainly the energy minimization back end. Shape currently relies on MM3 for the energy calculations and geometry relaxation. Thus it cannot process molecules that the MM3 back end have problems handling in an automated fashion. Some of the limitations of the MM3 package are overcome by the MM3-java package that is included in Shape.   
Development is under way to support back end software other than MM3, but the current version of Shape relies solely on MM3.  
  

### Installing Shape

Hopefully someone has already installed Shape for you. Then you can just use it as shown above. Otherwise you need to set it up. Shape is written primarily for \*nix systems such as linux, bsd, solaris, etc. It has not been tested for mac osx or windows yet, because the developers don't have access to an MM3 back end for these systems.   
To install Shape you need to know a few things about your computer environment.  

- You need to have java version 1.6 or newer installed on your system to run Shape. This should not be a problem since java 1.6 is quite old by now.
- You also need an installation of MM3 present on your system. An old MM3(92) package is provided in the Shape installation archive, and will be installed in a subdirectory under the Shape install directory. Shape also works with newer versions of MM3.
- If you will be using your own version of MM3, then you need to know where your MM3 installation can be found on your system, and what the MM3 binary and specific parameter and constants files are called. If you will use the old MM3(92) version that comes with the Shape package, then that has been taken care of and is already prepared in the shape.mm3.config file which describes the MM3 environment Shape is to use.

Start by unpacking the Shape.tbz2 archive file where you want Shape installed. It will unpack the Shape software package into a directory called "shape", and will place no files outside this location.   
Remember to change the ownership and read/write/execute permissions properly for the "shape" launch script, the source, output, error, and temporary directories. Don't forget to check the permissions of the MM3 directory and MM3 executables and data files as well.  
In some environments it might be a good idea to create a group named shape and allow all colleagues permissions to that group, then set up the permissions of the shape package to belong to that group.   
Once Shape is installed it is strongly recommended that you set up your system to mount a ramdisk for temporary work file storage. Then change the temporary and work directories in the configuration to point to directories located on ramdisk. This will improve Shape performance.   

##### Starting Shape

Once the Shape package is unpacked and permissions are correct you can set up your system to start the shape daemon automatically when the machine starts. That way it is always ready to process whatever new molecule files are put into the source directory.   
It is also possible to start Shape manually by running the "shape" launch script.   
If you start shape automatically it is recommended that you start it with a tool like
"screen", where it is possible to always log onto the "shape terminal session" to see what is going on in case something strange happends. Alternatively it can be a good idea to redirect the output to a log file if no terminal session is kept open.   
  
  
(fig 3) This is the typical look of the shape package, and initial output when starting the shape daemon without any molecule files in the source directory. The first command "ll" lists the typical content of the shape distribution archive. The second command "shape" starts the Shape daemon launch script and after performing basic checks Shape settles down, waiting for molecule files to appear in the source directory "src".  
  
If you start shape from the command line you can also give it some command line parameters for quick behaviour instructions. Run the provided shape launch script with flag "-h" or "--help" to see the basic usage.  
shape [configFile [srcDir [outDir [tmpDir [errDir]]]]]  
This means that you can direct Shape to run with different configuration files and directories by instructing it from the command line. For example:  
shape quicksearch.config  
will start shape using the configuration found in the "quicksearch.config" configuration file. This provides a quick and simple way to change the behaviour of Shape.  
  

##### Change the launch script

The launch script "shape" is an example for how to start shape and will work fine without changes. An alternative launch using the "screen" virtual terminal session tool is available in the script, but commented out. For more information on screen, see:
http://www.gnu.org/software/screen/   

### Configuration files

The Shape program package is controlled through the \*.config files. These are the files that contain all the configurable parameters that influence how the Shape program behaves when processing molecules. Below are links to information on the configuration parameters of each configuration file. It is, however, recommended that you start by reading the section on general configuration before starting to delve into changing the specific configuration files.   
**shape.config** is the primary configuration file for the shape package. It mainly describes file locations and work directories.   
**shape.server.config** is the configuration for the job distribution server. This specifies how many concurrent jobs should be run and temporary work locations.   
**shape.search.config** contains the parameters for the behaviour of the genetic algorithm that performs the conformation search.   
**shape.cluster.config** determines how the clustering of results should be done.   
**shape.mm3.config** describes the MM3 environment Shape will use for the energy minimizations during the search.   

### Monitoring Shape progress

Shape continually prints current status and progress to stdout. Thus it is simple to see what is going on and what it is doing. Simple logging is provided if you run Shape from a tool like screen and turn on logging, or by redirecting output from the screen process to a file.   
  
(fig 4) The complete output from a very quick search of a disaccharide file named "disacch.mol". When the "disacch.mol" source file is copied into the source directory Shape first verifies the file. Then follows the search evolution, which terminates after five generations. When the search is complete Shape clusters the results, finding eight clusters. Shape then goes back to sleep when it cannot find any more files in the source directory.  
  

### Bugs and Errors

Since most of the Shape package is prototype quality software there will be bugs. There are no known open bugs at this development snapshot, but there are most likely lots of bugs that the developers are not aware of. Hopefully, with your help, we can find more and eliminate them. If you see lots of strange messages in the Shape stdout and stderr output then it is likely that the program has crashed for some reason and is not behaving well any more. Some internal exceptions can be handled by the program and Shape will resume normal operation once it has taken care of the problem. If it does not, however, then try the steps below:  
1) Try to see if it is still running or not, i.e. Is it producing new status output, has it crashed to the command prompt, is the cpu still loaded with MM3 processes, are new files being created, etc.  
2) If it is not running properly then check the last input file for possible problems. Break and restart Shape for resuming work with the next molecule file. The most common source of problems is broken or unexpected input. If Shape finds fault with input files or configuration files then it will likely not behave well. Some "reasonable" level of resilience to strange input has been developed, but this is rather minimal since there are still quite few test users.   

### Filing bug reports

If you have found a bug, then contact the developers. But first, please try to reproduce the bug. See if you can get the program to crash the same way twice in a row, or produce the same error twice in a row. A reproducible bug is generally much easier to track down and fix. Especially if the developers can reproduce it on their own machines.   
If the developers can not reproduce the bugs on their machines but you can do so on yours, then please consider letting the developers have access to your machine via ssh for a little while, so that they more easily can see what the problem is and fix it. Intermittent bugs, problems that only show up every now and then, are generally much more difficult to fix.   
  
 **Make a good bug report:**   
1) Explain what the problem is.  
2) Describe how the program should behave when correct.  
3) Any specific settings and circumstances when the bug appears.  
4) Attach all configuration files and molecular source files that are required to reproduce the bug.   
  
Then send an email with this information to:  
email: jimmy rosen gmail com (insert "." and "@" at appropriate places)
