# Supplementary material for: Shape: automatic conformation prediction of carbohydrates using a genetic algorithm
Source: J Cheminform. 2009 Sep 21;1:16. doi: 10.1186/1758-2946-1-16 (PMC2820494; doi:10.1186/1758-2946-1-16)
Supplement: Additional file 1 — Shape version 090213. The complete shape distribution. [file 1758-2946-1-16-S1.TGZ › shape.release.090213/manual/mm3.config.html]

# Shape MM3 configuration

The MM3 configuration file specifies the MM3 environment that will be used for energy calculations and geometry minimizations. The default name of this file is "shape.mm3.config", but it can be changed in the main shape config file, commonly named "shape.config". Some of these values differ between MM3 versions, and changing them requires that you have some experience with your specific version of MM3. The default configuration provided with Shape is for the MM3(92) version that was used primarily for the Shape development.   
  
Parameter value pairs in this text are marked in  **bold monospace**  to make them easier to see.  
As usual with the shape configuration files all values are case sensitive. Parameters and values should be separated by spaces. Lines beginning with "#" hashmarks are treated as comments and ignored by Shape.  
  
  
The "rootDir" parameter specifies the root directory of all the MM3 related files and directories, i.e. the MM3 binary, constants and parameter files, work directory, output files, etc. The "rootDir" directory will be used as parent for any non rooted directory or file specified in the rest of the configuration file.   
 **rootDir /path/to/mm3**    
  
The "workDir" is where all temporary files go. It is recommended that you set it to a ramdisk for speed. Also, if running on a cluster, don't set this to point to a network mounted area, since then you'll be transporting lots of temporary data across the network to no use.   
 **workDir /ramdisk/shape.mm3.tmpworkdir**    
If workDir is not specified it will default to the present working directory.   
  
The "mm3Command" is the raw binary MM3 executable file that will be used to run MM3 calculations. This should not point to a wrapper script or suchlike.   
 **mm3Command mm392linux.no.static**    
  
The "paraOrgFile" and "constOrgFile" files are the "original" files for parameters and constants, provided with MM3.   
 **paraOrgFile para.org**    
 **constOrgFile const.org**    
  
MM3 require its execution input files to have certain names and be found in the execution work directory. These files will written in the workDir where MM3 is executed. The para and const files will be soft linked to their original files instead of copied and written for each invocation.   
 **cpdFileName CPD.MM3**    
 **constFileName CONST.MM3**    
 **paraFileName PARA.MM3**    
 **ta4FileName TAPE4.MM3**    
 **ta9FileName TAPE9.MM3**    
 **errFileName ERRMSG.MM3**    
  
These following open and close strings are markers used to locate the steric energy values in the tape4 files. They differ from different versions of MM3. The open and close values will surround the energy value, e.g.:   
 FINAL STERIC ENERGY IS 23.44 KCAL    
i.e. the program will match the pattern:   
 finalStericEnergyOpen floatvalue finalStericEnergyClose   
The two parameters are named "finalStericEnergyOpen" and "finalStericEnergyClose".   
 **finalStericEnergyOpen FINAL STERIC ENERGY IS**    
 **finalStericEnergyClose KCAL**    
  

Each block in an MM3 TAPE4 file is marked by a end of block marker. This is the string marker that marks the last line of a TAPE4 block. The parameter is named "ta4BlockEnd".   
 **ta4BlockEnd THIS JOB COMPLETED AT**
