# Supplementary material for: Shape: automatic conformation prediction of carbohydrates using a genetic algorithm
Source: J Cheminform. 2009 Sep 21;1:16. doi: 10.1186/1758-2946-1-16 (PMC2820494; doi:10.1186/1758-2946-1-16)
Supplement: Additional file 1 — Shape version 090213. The complete shape distribution. [file 1758-2946-1-16-S1.TGZ › shape.release.090213/manual/results.html]

# Managing Shape results

The results of each conformation search are written to a directory with the same name as the source molecule, located directly below the specified output directory, normally "out".  
  
The most interesting thing to look at is a quick view of the best ten or so cluster centroids, since they will represent the best local minima that were found in the conformation search. One way to do that is like this:  

```
    > pymol cluster.result/cluster.centroids/pdb/c*[0-9].*.pdb
```

which will open the ten best cluster centroids in the pymol viewer for an easy quick comparison of conformations. Replace with whatever software you use of course.  
  
The second most interesting thing to find in the cluster.result directory is the index of the cluster.centroids directory which tells you the relative energies and similarities of the local minima low energy conformations:

```
    > nedit cluster.result/cluster.centroids/index
```

which will open the nedit editor and show the index file. Replace with whatever software you use of course.   
  
It is recommended that you archive the results of each molecular search when you are finished with the results since Shape tends to write enormous amounts of small files, which can otherwise slow down some operations on your file system. The results are quite compressable, so tar archiving and bzip2 compression, or some such archiving scheme is recommended. An automated compression archiver is considered for later versions of Shape, but of low priority.   
  

### The source molecule directory

All the results are stored under a directory named the same as the original source molecule file. In this directory you will find:  

- The original molecule source file.
- The index of all generated conformations, named "index"
- The index of the search evolution, named "evolution".
- The directory named "all", which contains all generated individual conformations.
- The directory named "broken", which contains all broken files, if there were any which for some reason the evaluation back end could not manage.
- The directory named "cluster.results" which contains the results of the clustering operation.

  
The **index** file is the index of all generated individuals through the conformation search. It contains three columns.

- Column one is the id number of the individual conformation.
- Column two is the conformational energy of the individual conformation.
- Column three is the file name of the individual conformation. All individual files can be found in the "all" directory.

  
The **evolution** file is the index of the conformation search evolution. This file contains a trace of the evolution progress, showing how the average and best energy is improved through the search and which individuals belong to which population and generation.   
Each line in the file represents one generation for one population. Each line has five major columns.

- Column one is the population identifier.
- Column two is the generation identifier.
- Column three is the best energy found in that generation and population.
- Column four is the average energy of all individuals in that generation and population.
- Column five is a list of the identifiers of all individuals in that generation and population.

  
The **all** directory contains all individual conformations which has been generated and successfully minimized through the entire conformation search. The individuals are saved in an internal binary format. It is possible to convert these files to .pdb if you want, but it is simpler to use the results from the clustering process, which are already stored as .pdb files.  
  
The **broken** directory contains all individual conformations which turned out to generate errors in the search process, usually because of problems with MM3. This directory is almost always empty. If you find lots of files here there is something wrong, and you should contact the developers.  
  

### Results from the Clustering

The **cluster.result** directory contains all results from the clustering process, and this is where you will most likely find the interesting data from your search.  
The directory contains:

- A copy of the original clustering configuration file, for posterity.
- The clusterindex file, which lists all clusters sorted by lowest conformational energy of the cluster centroid.
- The file clusterindex.bySize, which lists all clusters sorted by size.
- The directory cluster.centroids, which contains copies of all the local minimum energy conformations, i.e. the cluster centroids.
- All the cluster directories, which are named cluster.x, where x is the identifier number of the cluster.

  
The **clusterindex** and **clusterindex.bySize** files have the same content, just ordered differently. The clusterindex has the clusters sorted by lowest energy, and the clusterindex.bySize have the clusters sorted by largest size.  
Both files have four major columns  

- Column one is the cluster identifier.
- Column two is the number of individuals in that cluster.
- Column three is the energy of the centroid of that cluster, i.e. the lowest conformational energy of all the individual conformations in that cluster.
- Column four is a list of all individuals in that cluster, sorted by lowest energy first.

  
The **cluster.x** directories contain all the individuals of that cluster converted to .pdb files, along with an index of the cluster.  
The **pdb** directory simply contains all individual conformations of the cluster saved as .pdb files.  
The **index** file of the cluster contains all individuals in the cluster along with their energy and the atom position root mean square deviation in Ångström, and not the clustering distance function. This measurement is used as a baseline to compare the distances within clusters when grouped as determined by the clustering configuration file.  
The index file has one individual per line and each line has three columns:

- Column one is the identifier of the individual.
- Column two is the energy of the individual.
- Column three is the atom position rmsd between this individual and the cluster centroid.

  
The **cluster.centroids** directory contains all cluster centroids converted to pdb files, along with an index of the cluster centroids.  
The **pdb** directory simply contains all cluster centroids saved as pdb files, and named in a way such that they will generally be listed alphabetically as lowest energy first, since the clusters are ordered by increasing energy.  
The **index** file of the cluster.centroids directory lists one centroid on each line, ordered by lowest energy first. Each line has five columns:

- Column one is the cluster identifier.
- Column two is the individual conformation identifier.
- Column three is the energy of that conformation.
- Column four is the atom position root mean square deviation between that individual and the lowest energy individual found in the entire search, i.e. between this centroid and the best one.
- Column five is the file name of the cluster centroid .pdb file.
