# Supplementary material for: Shape: automatic conformation prediction of carbohydrates using a genetic algorithm
Source: J Cheminform. 2009 Sep 21;1:16. doi: 10.1186/1758-2946-1-16 (PMC2820494; doi:10.1186/1758-2946-1-16)
Supplement: Additional file 1 — Shape version 090213. The complete shape distribution. [file 1758-2946-1-16-S1.TGZ › shape.release.090213/manual/search.config.html]

# Shape search configuration

This is the configuration for the Shape genetic algorithm search. It specifies how the genetic algorithm will behave when searching for the minimum energy conformation.   
Parametrization of a genetic algorithms (GA) is unfortunately a black art in itself. The values provided with the default configuration are supposed to do a reasonable trade-off between accuracy vs speed for carbohydrates of up to a few hundred atoms. The settings give an unnecessarily thorough search for smaller carbohydrates, which wastes some time. At the same time they are not accurate enough when the molecules are larger than a few hundred atoms.   
A tool for automatic parametrization of the GA to to better suit the target molecule at hand is planned, but delayed for more pressing work.   
  
  
Parameter value pairs in this text are marked in  **bold monospace**  to make them easier to see.  
As usual with the shape configuration files all values are case sensitive. Parameters and values should be separated by spaces. Lines beginning with "#" hashmarks are treated as comments and ignored by Shape.  
  
  
The populationSize parameter decides how many individuals there will be in each population throughout the search. A higher number gives a more thorough search, a smaller number gives a faster search.   
 **populationSize 20**    
  
The nrPopulations parameter decides how many parallel populations will be used during the search. A higher number gives a more thorough search and a smaller number gives a faster search.   
 **nrPopulations 10**    
  
In each generation there will be a total number of individuals equal to populationSize \* nrPopulations. The reason to allow for splitting the total number of individuals in each generation into smaller populations is to allow for a wider genetic diversity through the search. If only one population is used, then it is more likely that the search will get stuck in a local energy minimum, and will thus never find the global minimum.   
The current GA code only allows for generational evolution. Support for steady state evolution is considered for future releases.   
Crossover between populations is currently not enabled, although supported. For it to be really useful it requires checks for supressing similarity between populations, so that too similar individuals do not completely take over the global population. This is in development.   
  
The evolutionOperator parameter selects which evolution algorithm should be used.  
There is currently only one evolution operator supported; the SimpleEvolution class. The full qualifier is:  
  com.csol.chem.search.ga2.SimpleEvolution   
The evolutionOperator parameter requires a nested configuration for the class chosen.   
 **evolutionOperator com.csol.chem.search.ga2.SimpleEvolution {}**    
The {} should contain the configuration block for the SimpleEvolution class. More about that later.  
  
The convergenceCriterium parameter determines when the search will be terminated. There is currently only one convergence criterium enabled in this release;  
  com.csol.chem.search.ga2.EnergyWindowConvergence   
The class "EnergyWindowConvergence" terminates the search when a certain number of generations no longer find any significant improvements in conformational energy.  
 **convergenceCriterium com.csol.chem.search.ga2.EnergyWindowConvergence {}**    
The {} should contain the configuration block for the EnergyWindowConvergence class. More about that later.  
  

### com.csol.chem.search.ga2.SimpleEvolution configuration block

The SimpleEvolution operator class takes several configuration parameters that determine how the evolution should progress. The values provided in the default configuration file are reasonable for most molecules of the size for which the default configuration is targeted, but less efficient for very small and very large molecules.   
  
The parameter nrAncients specifies how many "ancient" individuals are allowed in a population. An ancient individual is one who does not undergo mutation and is kept for genetic "memory". It is the best individuals that are kept as ancients.  
The nrAncients should be set to either 0 or 1 in this release. A value higher than 1 will result in a too homogeneous population very fast. In later releases the CloneKiller feature will probably be enabled, which will eliminate individuals that are too similar, and then a higher nrAncients value might be beneficial. At current state it is recommended to be set to 1.  
 **nrAncients 1**   
  
The parameter useRankFitness decides whether the SimpleEvolution operator should use "rank fitness" or direct "energy fitness". Rank fitness assigns fitness based on which rank, i.e. place in a list sorted by energy, an individual conformation has. Direct energy fitness assigns fitness based on the raw energy values. Rank fitness has been shown to perform significantly better, and is thus strongly recommended for use here.  
 **useRankFitness true**   
The useRankFitness parameter takes the values true or false.  
  
If useRankFitness is set to "true" then some other parameters need to be set. This part of the configuration will change in the future to instead use a fitnessOperator, which is then specified just like the evolutionOperator and the convergenceOperator.  
The direct energy fitness is still enabled in this release for testing purposes, but will not be covered here.  
  
The parameter adjustRankFitnessRBySize should most likely be set to "true" since that will work best with variable size populations. You can turn it off, "false", if you know what you are doing and want to have good control of the ranking and fitness probabilities.   
If this parameter is on, "true", then the rank fitness ratio, i.e. the point in the rank list at which the fitness breeding selection probability is 50% of the top ranking individual, will be adjusted by the size of the population. This generally has the effect of enabling more genetic variation in the population. A higher effective rank fitness ratio will yield larger genetic variability.  
 **adjustRankFitnessRBySize true**   
The adjustRankFitnessRBySize parameter takes the values "true" or "false".  
  
The rankFitnessRatio parameter specifies the factor which decides at which point in the rank list the 50% of top rank breeding selection probability occurs. If you for example have a population of 20 individuals, and a rankFitnessRatio of 0.2, then the 50% mark will be associated by the fourth individual in the fitness ranking list. A higher effective rank fitness ratio will yield larger genetic variability.   
 **rankFitnessRatio 0.2**   
  
The rankFitnessRmin is the minimum rank fitness ratio index that will ever be used. This is useful if you have a mix of small and large populations. It sets the bottom limit of the breeding selection genetic variability. So, for example, even if you have only 10 individuals in a population and the rankFitnessRatio is 0.2, then the 50% mark should be at individual two in the fitness ranking list, but will be at individual tree, since that is the lower limit of the rank fitness ratio index set here.   
 **rankFitnessRmin 3**   
  
The rankFitnessRmax is the corresponding maximum rank fitness ratio index that will ever be used. This is useful for the large populations, where too high variability would lead to very slow convergence.   
 **rankFitnessRmax 10**   
  
If you do not want to use automatically adjusted rank fitness values, then you can instead turn off the adjustRankFitnessRBySize by setting it to "false", skip the rankFitnessRatio, rankFitnessRmin, and rankFitnessRmax, and instead provide the direct probability and rank index by the two parameters: rankFitnessP and rankFitnessR. These should not be specified if automatic adjustment by population size is turned on; adjustRankFitnessRBySize "true".  
 **rankFitnessP 0.5**   
 **rankFitnessR 3**   
Here the breeding selection fitness probability 0.5 (50%) will be assigned to the individual at fitness rank list index 3.  
  
The last parameter of the SimpleEvolution evolutionOperator is the mutationOperator, which is in itself a configuration block, although a very simple one. The mutationOperator decides how to mutate new individuals. That means how much they should be mutated and how significant each mutation should be.   
Currently there are two possible mutationOperators available: MutateByRatio and MutateByRate. The MutateByRatio mutationOperator mutates the individual by a certain fraction of the genome, while the MutateByRate mutationOperator considers each gene by itself and has a probability of mutating each individual gene.  
 **mutationOperator com.csol.chem.search.ga2.MutateByRatio {}**   
Where the {} block should be replaced with the configuration block of the selected mutationOperator.  
  
Future versions of the SimpleEvolution evolutionOperator will contain the configuration blocks for the crossoverOperator as well. At current development status this is still undergoing change and testing. It is currently default configured by the program to the best values currently known. Once the behaviour and impact is better understood the important configuration values will be made open to the end user for change.   
  

### com.csol.chem.search.ga2.MutateByRatio configuration block

The com.csol.chem.search.ga2.MutateByRatio mutationOperator takes just one parameter; the mutationRatio, which is specified as a probability and decides the fraction of the genome to be mutated. A value of 0.05 means that 5% of the genome will be mutated. The MutateByRatio mutationOperator will however always mutate at least one gene.   
 **mutationRatio 0.05**   
  

### com.csol.chem.search.ga2.MutateByRate configuration block

The com.csol.chem.search.ga2.MutateByRate mutationOperator takes just one parameter; the mutationRate, which is specified as a probability and decides the rate of mutation. Each individual gene has this specified probability to be mutated. A value of 0.05 means that each gene has a 5% chance of being mutated. The MutateByRate mutationOperator is guaranteed to always mutate at least one gene.   
 **mutationRate 0.05**   
  

### com.csol.chem.search.ga2.EnergyWindowConvergence configuration block

The EnergyWindowConvergence convergenceOperator takes two parameters; window and limit. These values determine how persistent the search engine should be before giving up and accepting the results as the "best that can be found" in this specific search run.  
  
The window parameter determines the size of the window through which to search for improvement. This is directly related to maximum efficiency of the evolution, since the window size is the absolute minimum limit to the length of the search. Once the "best" conformation has been found, the search will always continue to search for a number of generations equal to the window size, before it is satisfied that it has converged.  
 **window 10**   
  
The limit parameter is the highest energy difference throughout the window that is accepted as improvement significant enough to continue the search. This is calculated as the value of Em - E0, where E0 is the energy of the oldest individual in the window, and the Ex is the lowest energy (best individual) in the window. Thus, the maximum value that can be used for the limit parameter is 0.0, since that denotes that any improvement, however miniscule it may be, is important enough to continue the search. A more reasonable value is -0.5, which says that if the search hasn't produced anything better than -0.5 kcal energy difference over all the generations in the window, then it can stop.  
The lower the limit, the faster the search will give up. Observer however that -1.0 is lower than -0.5 and 0.0, since these are signed, not absolute values.   
 **limit -0.5**
