# Supplementary material for: Shape: automatic conformation prediction of carbohydrates using a genetic algorithm
Source: J Cheminform. 2009 Sep 21;1:16. doi: 10.1186/1758-2946-1-16 (PMC2820494; doi:10.1186/1758-2946-1-16)
Supplement: Additional file 1 — Shape version 090213. The complete shape distribution. [file 1758-2946-1-16-S1.TGZ › shape.release.090213/manual/server.config.html]

# Shape server configuration

Shape server configuration determines how jobs should be distributed and how many jobs should be run in parallel. The server configuration defaults to the name "shape.server.config" but it can be changed form the main shape config file, normally "shape.config".   
The only server subsystem available in this release is the MM3 cluster/SSI/SMP server that was originally developed for OpenMosix clusters. It can distribute jobs on multi cpu / multi core systems and clusters that support process migration.   
  
  
Parameter value pairs in this text are marked in  **bold monospace**  to make them easier to see.  
As usual with the shape configuration files all values are case sensitive. Parameters and values should be separated by spaces. Lines beginning with "#" hashmarks are treated as comments and ignored by Shape.  
  
  
The "rootDir" parameter is used as root for all non rooted file paths.   
 **rootDir /home/tools/shape**    
  
The nrClients parameter specifies how many clients should be instantiated and used to run jobs. This should be equal to or slightly higher than the number of physical thread execution units in your computing system architecture. For a common dual core machine the number should be 2, for a dual cpu hyperthreaded machine it should be 4, for a cluster of machines just sum up all the cpus/cores/hyperthreads/etc.   
 **nrClients 2**    
  
The work directory where temporary files are written. This is recommended to reside on ramdisk for better performance. This is the temp work for the distributed jobs. It can be different than the temporary work directory specified in the main Shape configuration file, but in most cases it should be the same. When running on clusters with network distributed filesystems it can make sense to have different work areas. Normally it should be the same.   
 **workDir /ramdisk/shape.server.work**    
  
The Shape MM3 configuration to be used for running the distributed jobs. This can differ from the MM3 configuration provided in the main shape configuration file, but should be the same in most circumstances. The primary reasons for running with different configurations for server and main Shape MM3 validation is when running on clusters and network distributed filesystems. Normally it should be the same.   
 **mm3ConfigFile shape.mm3.config**
