# Supplementary material for: Shape: automatic conformation prediction of carbohydrates using a genetic algorithm
Source: J Cheminform. 2009 Sep 21;1:16. doi: 10.1186/1758-2946-1-16 (PMC2820494; doi:10.1186/1758-2946-1-16)
Supplement: Additional file 1 — Shape version 090213. The complete shape distribution. [file 1758-2946-1-16-S1.TGZ › shape.release.090213/manual/index.html]

# Shape manual

introduction  
  
configuration  
  
results  
  
output

Above are the basic areas of interest for setting up and running Shape. The Introduction will provide the basic user information, and the first few paragraphs of that section is usually enough to actually use Shape.  
Setting up Shape, or changing its behaviour is somewhat more complex. Setup is covered in the introduction. Configuration is covered in the configuration section, which also links to subsections on all the configuration files that Shape needs to function.  
The results section describes in detail all the files and directories that will be produced as actual results of a conformation search. Usually you don't need all this, just some basics which are already described in the introduction section.  
Shape output, e.g. status reporting, consol chatter, is described in the output section.
