# Supplementary material for: Shape: automatic conformation prediction of carbohydrates using a genetic algorithm
Source: J Cheminform. 2009 Sep 21;1:16. doi: 10.1186/1758-2946-1-16 (PMC2820494; doi:10.1186/1758-2946-1-16)
Supplement: Additional file 1 — Shape version 090213. The complete shape distribution. [file 1758-2946-1-16-S1.TGZ › shape.release.090213/manual/cluster.config.html]

# Shape clustering configuration

The clustering tool is the most varied of the shape tools in this release, as it has plenty of operator modules which can be switched in and out to provide the clustering behaviour the user wants. The downside to this is that the configuration is more complex.   
Basically, for the current release the clustering algorithm itself is rather simple, and the complexity of the configuration comes from the multitude of modules that change measurement behaviour.   
Clustering can be very time consuming if done thoroughly, which is why the clustering algorithm implemented here is rather limited. It is, however, well suited to the task at hand. The basic need for clustering in the Shape package is to cluster similar conformations around the local low energy minima, to make it easier to get a useful overview of the search results.   
  
A fast hierarchic clustering function and a reduced dimension geometry hash based clustering function are considered for future development, but of low priority at the moment. If you feel the primary clustering function is of too poor quality then please notify the Shape developers, or you can take a look at the source and write one yourself.  
The API is trivial, and using this reflective configuration your clustering code can be directly plugged into the Shape program without touching any other Shape code outside your new clustering class.   
see: com.csol.chem.util.analysis.LECFunction.java for the example implementation. It is only about 25 lines of code logic. The interface is: com.csol.chem.util.analysis.ClusteringFunction.java   
This also applies if you want to include other measurement or weight functions. Most of the classes are quite small and simple.  
  
Execution time for clustering in this release is dependent upon:

- Number of atoms in the molecule. The alignment operators have an O(n^2) complexity to the number of atoms in the molecule.
- Number of conformations. The clustering algorithm normally operates in approximately O(n^1.5) complexity to the number of conformations to cluster.
- Tolerance of the clustering. The clustering complexity is inversely correlated to the tolerance. Higher tolerance will yield faster clustering.

  
Parameter value pairs in this text are marked in  **bold monospace**  to make them easier to see.  
As usual with the shape configuration files all values are case sensitive. Parameters and values should be separated by spaces. Lines beginning with "#" hashmarks are treated as comments and ignored by Shape.  
  
  
The only base level parameter in the clustering file is the clusteringFunction parameter. The only clustering function in this release is the com.csol.chem.util.analysis.LECFunction. The LECFunction simply grows cluster around local minima, up to a certain tolerance. This is fast and simple, but gives quite good results in practice.   
 **clusteringFunction com.csol.chem.util.analysis.LECFunction {}**   
The LECFunction requires a block configuration and the {} should be expanded to include the complete configuration block for the LECFunction class.   
  

### com.csol.chem.util.analysis.LECFunction configuration block

The LECFunction clusteringFunction takes two parameters. These are the tolerance and the distanceFunction. The tolerance is the maximum distance from the cluster centroid at which a conformation will be considered as part of the cluster.  
The unit of distance will be different depending on how the distance is measured, and several different measurements are possible. In the configuration example below it is stated as a 1.0 Ångström RMSD based on atom positions. It can also be a measurement in degrees, if the distanceFunction selected is a torsion angle based distanceFunction operator.   
 **tolerance 1.0**   
  
The distanceFunction determines how the distance between two conformatons is measured. There are two distanceFunction operator classes available in this release:  
com.csol.chem.util.analysis.AtomRMSD  
com.csol.chem.util.analysis.TorsionAngleRMSD  
These two distanceFunctions will measure either the atom position RMSD or the internal torsion angle RMSD between conformers.  
 **distanceFunction com.csol.chem.util.analysis.AtomRMSD {}**   
Both the AtomRMSD and TorsionAngleRMSD distanceFunction operator classes requires configuration blocks, and thus the {} should be expanded to include the complete configuration for these classes.   
  

### com.csol.chem.util.analysis.AtomRMSD configuration block

The AtomRMSD distanceFunction operator measures the root mean square deviation of the atom positions between two conformations. The unit is Ångström.   
Both the AtomRMSD and the TorsionAngleRMSD distance functions take only one parameter; the weightFunction operator. The weightFunction operator decides what weight (importance) is to be given to different distance properties of the conformations. One example is to put a higher importance (weight) on the heavier atoms, ignoring hydrogens.   
There are several different AtomRMSD weightFunction operators available in this release:  
com.csol.chem.util.analysis.AtomWeightByMass
com.csol.chem.util.analysis.AtomWeightBySqrtMass  
com.csol.chem.util.analysis.AtomWeightHeavyOnly  
com.csol.chem.util.analysis.AtomWeightUniform  
You can of course also write your own and plug in if these are not enough. The code in these examples is short and trivial.   
 **weightFunction com.csol.chem.util.analysis.AtomWeightUniform {}**   
Each of the weightFunction operators require a configuration block {}, but in most cases this is just an empty block. The empty block is still expanded to two lines. The opening curly brace { is to be placed on the first line, directly behind the class qualifier, and the closing curly brace } should be placed on a line below. It is comment to place the comment "# takes no parameters" in the block to clarify that the block should be empty from parameters.  
  

### com.csol.chem.util.analysis.TorsionAngleRMSD configuration block

The TorsionAngleRMSD distanceFunction operator measures the root mean square deviation of the internal torsion angles between two conformations. The measurement is in degrees, not radians.   
Both the AtomRMSD and the TorsionAngleRMSD distance functions take only one parameter; the weightFunction operator. The weightFunction operator decides what weight is to be given to different distance properties of the conformations. Examples include putting a higher importance (weight) to the heavier atoms, ignoring hydrogens, or to make sure that torsion angles close to the center of the molecule are given stronger importance than those in the molecule's outer regions.   
There are several TorsionAngleRMSD weightFunction operators available in this release:  
com.csol.chem.util.analysis.TorsionAngleWeightBySubTreeMass  
com.csol.chem.util.analysis.TorsionAngleWeightByLnSTM  
com.csol.chem.util.analysis.TorsionAngleWeightBySqrtSTM  
com.csol.chem.util.analysis.TorsionAngleWeightHeavyOnly  
com.csol.chem.util.analysis.TorsionAngleWeightUniform  
You can of course also write your own and plug in if these are not enough. The code in these examples is short and trivial.   
 **weightFunction com.csol.chem.util.analysis.TorsionAngleWeightBySubTreeMass {}**   
Each of the weightFunction operators require a configuration block {}, but in most cases this is just an empty block. The empty block is still expanded to two lines. The opening curly brace { is to be placed on the first line, directly behind the class qualifier, and the closing curly brace } should be placed on a line below. It is comment to place the comment "# takes no parameters" in the block to clarify that the block should be empty from parameters.  
  

### AtomRMSD weightFunction operators list and their configuration blocks

The AtomRMSD weightFunction operator classes are used to assign a weight (importance) to an atom. The available ones are:  
  
**com.csol.chem.util.analysis.AtomWeightUniform**  
The AtomWeightUniform gives all atoms the same weight.  
  
**com.csol.chem.util.analysis.AtomWeightByMass**  
The AtomWeightByMass operator simply places weight importance on atoms based on their mass. Thus a Hydrogen will have approximately weight 1 and a Carbon approximately weight 12.  
  
**com.csol.chem.util.analysis.AtomWeightBySqrtMass**  
The AtomWeightBySqrtMass simply assigns weights based on the square root of the atom mass.  
  
**com.csol.chem.util.analysis.AtomWeightHeavyOnly**  
The AtomWeightHeavyOnly assigns the uniform weight of 1 to all atoms heavier than the specified cutoff mass, and all atoms below will be given the weight 0. Thus the AtomWeightHeavyOnly requires the parameter cutoffMass. A value of cutoffMass 2.0 will simply remove the importance of all Hydrogens from the distance measurements.   
 **cutoffMass 2.0**   
  

### TorsionAngleRMSD weightFunction operators list and their configuration blocks

The TorsionAngleRMSD weightFunction operator classes are used to assign a weight (importance) to a torsion angle.   
The available TorsionAngleRMSD weightFunction operators are:  
  
 **com.csol.chem.util.analysis.TorsionAngleWeightUniform**   
The TorsionAngleWeightUniform will assign the weight 1 to all torsion angles.  
  
 **com.csol.chem.util.analysis.TorsionAngleWeightBySubTreeMass**   
The TorsionAngleWeightBySubTreeMass will assign a weight to the torsion angle which is equal to the total mass of all atoms spanned by the smallest sub tree of the molecular graph separated by the torsion angle bond, and ignoring the two atoms of that bond.  
  
 **com.csol.chem.util.analysis.TorsionAngleWeightByLnSTM**   
The TorsionAngleWeightByLnSTM is similar to the TorsionAngleWeightBySubTreeMass, but instead of the full mass of the sub tree it will return the natural logarithm of the mass.  
  
 **com.csol.chem.util.analysis.TorsionAngleWeightBySqrtSTM**   
The TorsionAngleWeightBySqrtSTM is similar to the TorsionAngleWeightBySubTreeMass, but instead of the full mass of the sub tree it will return the square root of the mass.  
  
 **com.csol.chem.util.analysis.TorsionAngleWeightHeavyOnly**   
The TorsionAngleWeightHeavyOnly also calculates the total mass of the smallest sub tree of the molecular graph that is separated by the torsion angle bond. It will then return the weight 1 for all torsion angles which have a sub tree mass greater than the cutoffMass parameter, and a weight of 0 for all torsion angles with a sub tree mass smaller than the cutoffMass. This means that the TorsionAngleWeightHeavyOnly requires the parameter cutoffMass to be specified.   
For example, the value cutoffMass 2.0 will ignore all hydroxyl torsions, since the oxygen in those torsion angle definitions would not be included in the sub tree mass, as it is one of the two atoms of that torsion angle bond.  
 **cutoffMass 2.0**
