# Supplementary material for: Shape: automatic conformation prediction of carbohydrates using a genetic algorithm
Source: J Cheminform. 2009 Sep 21;1:16. doi: 10.1186/1758-2946-1-16 (PMC2820494; doi:10.1186/1758-2946-1-16)
Supplement: Additional file 1 — Shape version 090213. The complete shape distribution. [file 1758-2946-1-16-S1.TGZ › shape.release.090213/manual/shape.config.html]

# Shape main configuration

This is the basic configuration file for the Shape conformation search tool. The default name of this file is "shape.config", but it can be changed on the command line when starting the shape daemon.  
  
  
Parameter value pairs in this text are marked in  **bold monospace**  to make them easier to see.  
As usual with the shape configuration files all values are case sensitive. Parameters and values should be separated by spaces. Lines beginning with "#" hashmarks are treated as comments and ignored by Shape.  
  
  
The Root Directory "rootDir" is the base directory to which all other non rooted directories will be considered as relative to. E.g. if you have the root directory   
 rootDir /path/to/root   
and a relative (non rooted) directory   
 srcDir some/source/dir   
then the srcDir actually points to
 /path/to/root/some/source/dir   
A relative (non rooted) directory is any directory that does not start with a root qualifier, e.g. "/" on \*nix systems, and "C:", "D:", etc on windows systems.   
 **rootDir /home/tools/shape**    
The rootDir parameter defaults to the present working directory if not specified.  
  
The Source Directory "srcDir" is where the Shape program will search for molecular source files to perform conformation search on. All files in the source directory will be used for conformation search, and attended to in a first come first served order, based on the age of each file.   
 **srcDir src**    
The srcDir parameter defaults to "src" if not specified.   
  
The Output Directory "outDir" is where all results are written. Shape will create a directory for each source file under the output directory and write all results there.   
 **outDir out**    
The outDir parameter defaults to "out" if not specified.  
  
The Error Directory "errDir" is where all problematic source files are written. The user can then later go through them manually and see what might be the problem. If you cannot find a problem with the file, please report this as a bug and notify the Shape developers, please also include the problematic file in the message.  
Main developer: jimmy rosen gmail com (insert . and @ where appropriate)   
 **errDir err**    
The errDir parameter defaults to "err" if not specified.  
  
The Temporary work Directory "tmpDir" is where all incomplete, unimportant, temporary files are placed. It is recommended that this is placed in a ramdisk for quick access. It should normally be small, usually no larger than a few MB in size. If you run this over a cluster it is very important that this destination is on a local file system, otherwise a lot of data will run through the network for no good reason.   
 **tmpDir tmp**    
The tmpDir parameter defaults to "tmp" if not specified.   
  
The "mm3ConfigFile" points to the primary configuration file for the MM3 program that is to be used for energy calculations and geometry relaxation.   
 **mm3ConfigFile shape.mm3.config**    
The mm3ConfigFile parameter will default to "shape.mm3.config" if not specified.   
  
The "serverConfigFile" points to the primary configuration file for the job distribution server.   
 **serverConfigFile shape.server.config**    
The serverConfigFile parameter will default to "shape.server.config" if not specified.   
  
The "gaSearchConfigFile" points to the primary configuration file for the Genetic Algorithm search engine.   
 **gaSearchConfigFile shape.search.config**    
The gaSearchConfigFile parameter will default to "gaSearchConfigFile" if not specified.   
  
The "clusteringConfigFile" points to the primary configuration file for the clustering engine.   
 **clusteringConfigFile shape.cluster.config**    
The clusteringConfigFile parameter will default to "shape.cluster.config" if not specified.   
  
The "sleepInterval" is the time to sleep between looking for new files in the source directory "srcDir", if it was previously found to be empty. The value is specified in milliseconds [ms].   
 **sleepInterval 10000**    
The sleepInterval parameter will default to "10000" if not specified, i.e. 10 seconds.
