# Supplementary figures and images for: Shape: automatic conformation prediction of carbohydrates using a genetic algorithm
Source: J Cheminform. 2009 Sep 21;1:16. doi: 10.1186/1758-2946-1-16 (PMC2820494; doi:10.1186/1758-2946-1-16)

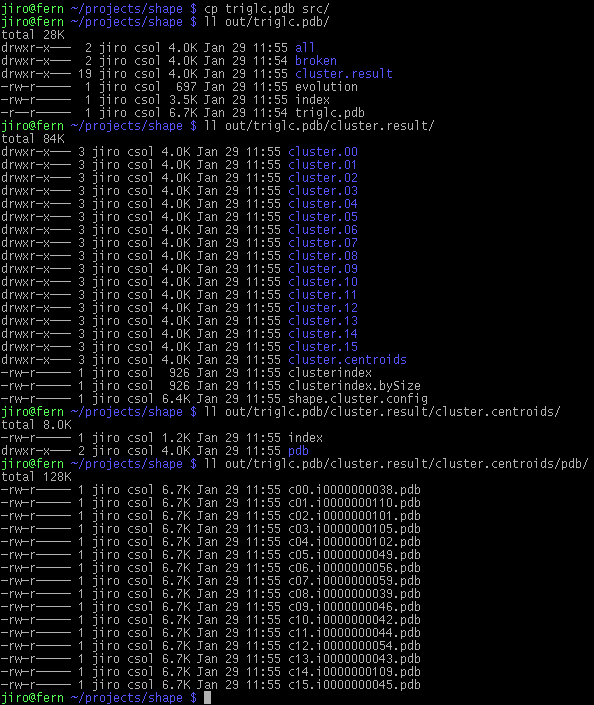

Supplement: Additional file 1 — Shape version 090213. The complete shape distribution. [file 1758-2946-1-16-S1.TGZ › shape.release.090213/manual/fig/triglc-session.png]

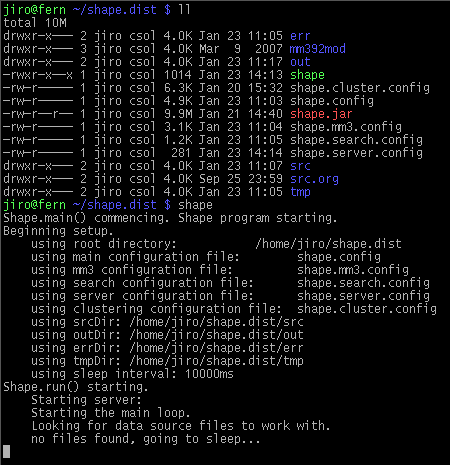

Supplement: Additional file 1 — Shape version 090213. The complete shape distribution. [file 1758-2946-1-16-S1.TGZ › shape.release.090213/manual/fig/shape-starting.png]

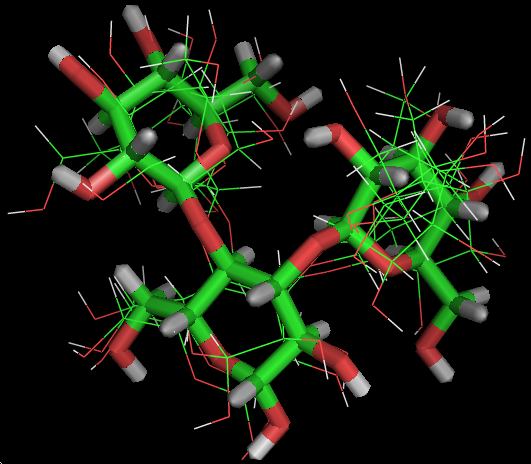

Supplement: Additional file 1 — Shape version 090213. The complete shape distribution. [file 1758-2946-1-16-S1.TGZ › shape.release.090213/manual/fig/pymol-6-clusters.png]

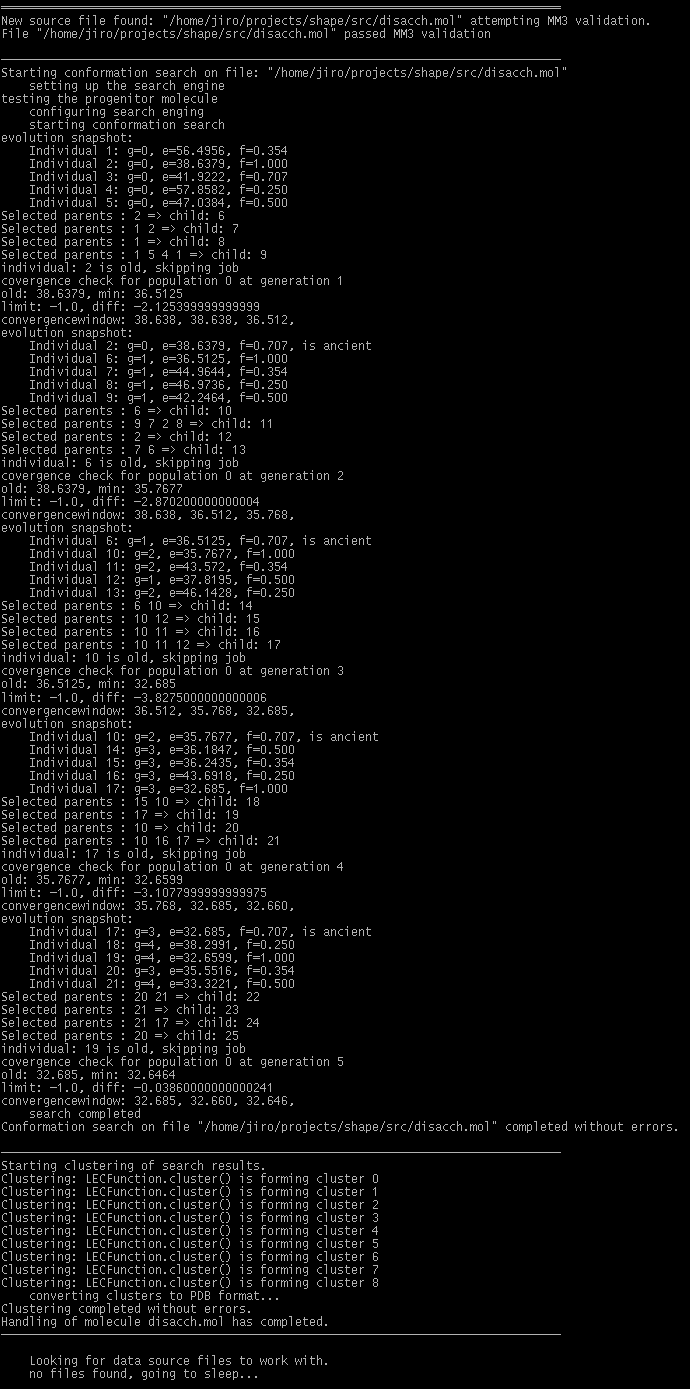

Supplement: Additional file 1 — Shape version 090213. The complete shape distribution. [file 1758-2946-1-16-S1.TGZ › shape.release.090213/manual/fig/complete-run.png]
